# Chloroplast symbiosis in a marine ciliate: ecophysiology and the risks and rewards of hosting foreign organelles

**DOI:** 10.3389/fmicb.2012.00321

**Published:** 2012-09-04

**Authors:** George B. McManus, Donald M. Schoener, Katharine Haberlandt

**Affiliations:** Department of Marine Sciences, University of ConnecticutGroton, CT, USA

**Keywords:** mixotrophy, *Strombidium*, oligotrich, chloroplast, photosynthesis, kleptochloroplast

## Abstract

Simultaneous use of both heterotrophic and autotrophic metabolism (“mixotrophy”) is common among protists. *Strombidium rassoulzadegani* is a planktonic mixotrophic marine ciliate that saves chloroplasts from its algal food and obtains a nutritional subsidy via photosynthesis. Cultures from the northeast, northwest, and southwest Atlantic Ocean show similar numerical response parameters (maximum growth rate, food concentration at which growth is half its maximum, and threshold food concentration for growth), and some isolates have been maintained *in vitro* for over 3 years. This ciliate grows equally well when fed on the green alga *Tetraselmis chui* (strain PLY429) or the cryptophyte *Rhodomonas lens* (strain RHODO). It appears to be an obligate mixotroph, requiring both food and light to achieve positive growth, when feeding on either of these algae. However, it has also been grown for several weeks (>10 generations) heterotrophically on the dinoflagellate *Prorocentrum minimum* (strain EXUV) during which it grows better in dark than in light. In this paper, we review the ecology of *S. rassoulzadegani*, discuss some aspects of its photo- and feeding physiology, and speculate on benefits and costs to the ciliate of chloroplast symbiosis.

## Introduction

Oligotrich ciliates are among the most abundant small zooplankters in the sea. As grazers of primary production and food items for metazoan plankton they constitute an important link in the ocean's planktonic food web (Perez et al., 1997; Smetacek, 1981; Verity et al., 1999; Dolan et al., 2002; Olson and Strom, 2002; Ota and Taniguchi, 2003; Urrutxurtu et al., 2003). Early observers of oligotrichs sometimes reported colored bodies or other algal-like inclusions that were assumed to be the remains of ingested food (Calkins, 1901; Lohmann, 1908; Kahl, 1930). With the advent of fluorescence and electron microscopy, however, it was noted that some oligotrichs contain intact chloroplasts from ingested algae (Laval-Peuto and Febvre, 1986; Laval-Peuto et al., 1986; McManus and Fuhrman, 1986; Jonsson, 1987; Stoecker et al., 1987; Laval-Peuto and Rassoulzadegan, 1988) and subsequent work has shown that these chloroplasts remain functional within the cytoplasm for days to weeks, providing a photosynthetic subsidy to the ciliates' metabolism (Jonsson, 1987; Stoecker et al., 1988b; Putt, 1990). This mode of living has been referred to as kleptoplasty (stealing chloroplasts), or chloroplast “symbiosis,” “sequestration,” “retention,” or “enslavement.” It represents a kind of mixotrophy (having both auto- and heterotrophic nutritional modes) and appears to be common in ciliate assemblages in both oligotrophic and eutrophic environments, with mixotrophs comprising roughly 30% of all ciliates in the plankton (Stoecker et al., 1987; Perez et al., 1997; Dolan et al., 1999).

Concomitant with the discovery that many oligotrich ciliates harbor intact chloroplasts retained from ingested food was the recognition that ciliates and other microzooplankton are important trophic intermediaries (Gifford, 1988, 1991; Montagnes et al., 1988). It was thus quickly appreciated that mixotrophic ciliate photosynthesis could be an important carbon flow in planktonic food webs of marine waters, especially that in the microplankton size class (Stoecker et al., 1987).

In a recent review, Stoecker et al. (2009) categorized most oligotrich ciliate mixotrophy as a “persistent/obligate” arrangement, i.e., for a given mixotrophic species, retained chloroplasts are almost always present, and the ciliate cannot grow in the dark or on a diet of aplastidic cells, even if food is plentiful (Stoecker et al., 1988b). Because the oligotrichs are in general fastidious organisms that are difficult to maintain in culture for long periods of time (Gifford, 1985), experimental approaches to understanding the incorporation, maintenance, and management of retained chloroplasts have been limited to short durations. Comprehensive studies of food preferences and contributions of photosynthesis to the ciliate energy budget have been limited [cf. review in Johnson (2011)]. In a number of cases, it has been established that incorporation of inorganic carbon may account for enough energy to meet the maintenance respiratory requirements of the ciliate (Stoecker et al., 1988b; Putt, 1990) but there are currently insufficient data to support this as a generalization.

Persistent/obligate chloroplast retention has been viewed as a kind of symbiosis in other protists and in metazoa (Trench, 1969; Lopez, 1979). In the sense of “living together,” the intact functional chloroplast and its ciliate host are indeed involved in a symbiotic relationship. However, since the chloroplast is from a genetically dead alga, its fitness when sequestered is zero and the relationship provides no apparent evolutionary benefit to the alga. From the point of view of the ciliate, however, the symbiotic nature of the relationship is apparent in the contribution of the chloroplast's photosynthesis to its metabolism. What is not apparent is whether there are any costs to the ciliate of this arrangement that would make it a true evolutionary tradeoff in the same way that maintenance of zooxanthellae by corals or other forms of symbiosis are (Muller-Parker and D'Elia, 1997; Yakovleva et al., 2009).

The purpose of this paper is to review some features of chloroplast retention in the oligotrich *Strombidium rassoulzadegani*. Unlike many of its fellow mixotrophic oligotrichs, this species is quite amenable to cultivation and isolates have been maintained in the laboratory for several years. We will review the distribution of this species, discuss its cultivation both hetero-and mixotrophically, and report on some aspects of its growth and photosynthesis. In this context, we will also speculate on the costs of this form of mixotrophy.

## Materials and methods

### Isolation and cultivation

*S. rassoulzadegani* is reliably found in tide pools in temperate regions, being most abundant during summer. It has grass-green chloroplasts. Usually, a prominent red eyespot located in an apical bump at the anterior of the cell can be observed. For isolation, we either picked individuals with a drawn capillary pipette or enriched with the green microalga *Tetraselmis chui* (strain PLY429). This ciliate grows well on f/2 medium (Guillard and Ryther, 1962) in six-well plates (volume about 7 ml) at a salinity of 30, temperature of 19°C and a 12:12 light:dark cycle at 50–100 μmol m^−2^s^−1^. It appears to be euryhaline and eurythermal (data not shown). For larger volumes, we used flat tissue culture flasks and maintained a level of medium that resulted in the same surface area to depth ratio as in the well plates. Every time we have brought this species into culture, we verified its identity by comparing its internal transcribed spacer (ITS) sequence to that of the original isolate. It is curious that despite the observed ITS diversity of green tidepool ciliates *in situ* (Katz et al., 2005), *S. rassoulzadegani* is the only one that has been successfully cultivated for any length of time, to our knowledge.

Growth at different food concentrations was evaluated by placing 10–15 ciliates from well-fed cultures into wells at various food levels, in triplicate. Ciliates were first acclimated to food concentrations for 12–24 h, then transferred to new wells at each concentration. Growth was measured as change in ciliate abundance over 3 days, assuming exponential growth. Numerical response curves (growth rate, μ, versus food concentration) were fit to a Michaelis-Menten function with the addition of a threshold parameter (food concentration at which net growth is zero; Montagnes et al., 1996):

$$\mu =\mu_{\text{max}}[\text{C-t}]/(\text{K+}[\text{C-t}])$$

where μ is specific growth rate (d^−1^), μ_max_ is the maximum growth rate, C is food concentration, K is the food concentration at which μ is half of μ_max_, and t is the threshold food concentration. Curves were fit iteratively using SigmaPlot software.

To evaluate the effects of light level on growth, we exposed cultures of *S. rassoulzadegani* to various light levels using a fluorescent light table and neutral density film. Cultures grown at 100 μmol m^−2^s^−1^ were transferred to wells at saturating food concentrations (2 × 10^4^ cells ml^−1^) and continuous light levels from 0 to 200 μmol m^−2^s^−1^. Growth was measured over 3d and calculated assuming exponential growth. There was no initial acclimation period at the lower light levels because the resulting slower growth would have differentially aged the ciliates' chloroplasts and we wanted the chloroplast status to be uniform across all light levels at the start of the experiment.

### Autotrophy

To evaluate inorganic carbon uptake, we adapted the ^14^C uptake method of Skovgaard et al. (2000). Ciliates that had been grown on the chlorophyte *Tetraselmis chui* (PLY429) were picked with a drawn pipette, rinsed in filtered seawater (FSW), and placed into six 20 ml scintillation vials containing 0.5 ml of FSW at a final count of 20 cells per vial. We also prepared six FSW controls and six vials of its food alga at 2 × 10^4^ cells ml^−1^ so that we could compare algal and ciliate photosynthetic rates. We then spiked the algae, algae-free ciliates, and FSW controls with NaH^14^CO_3_ to a target final activity of 0.5 mCi ml^−1^. To measure activity, 100 μ l samples were taken from the FSW controls before and immediately after the NaH^14^CO_3_ addition, added to 200 μ l of phenylethamine, an organic base, and measured with a liquid scintillation counter (LSC). Half of the experimental vials were incubated in the light (100 μmol photons m^−2^s^−1^) and half in the dark for 4 h. After incubation, vials were acidified and dried down to remove unincorporated inorganic carbon, leaving behind the carbon fixed by the ciliates. Samples were then re-suspended in 0.5 ml deionized water and activity was measured. The total inorganic carbon in the medium was determined using the method described in Parsons et al. (1984). Carbon incorporation rates (P) were calculated as

$$P=\frac{\left(DPM_{L}-DPM_{D}\right)\times W}{DPM_{t=0}\times t}$$

where P is in units of ngC ml^−1^ d^−1^, DPM are disintegrations per minute in light (L), in dark (D), and at initial time (*t* = 0); W is the inorganic carbon concentration in the medium, and *t* is incubation time. Knowledge of cell concentrations and chlorophyll and carbon content were used to convert to rates per cell and turnover (d^−1^).

### Phototaxis

To evaluate phototaxis, we used a modified flat capillary assay (Levandowsky et al., 1984). Ciliates fed for several days on various algae were picked by drawn pipette in batches of 10 and placed in capillaries 50 mm long by 8 mm wide by 0.4 mm deep. The capillaries were shaded for one half their length with aluminum foil. Ciliates were initially placed in the dark half of the capillary and the capillary was placed on the stage of a stereomicroscope with the substage light turned on. After 2 min, the proportion of ciliates that had migrated to the light was measured. The capillary was then turned end-to-end and the foil cover moved to the other half, returning the ciliates from the lighted half back to darkness for the next trial. For each food alga, we conducted five trials. Ciliates were also examined by compound light microscopy for maintenance of an eyespot on different foods.

### Short-term light/dark growth after acclimation

We performed several experiments to evaluate the degree to which mixotrophy is obligate in *S. rassoulzadegani* by comparing growth on several algal foods under light and dark conditions. In the first experiment, we compared growth on *Tetraselmis chui* (PLY429), *Rhodomonas lens* (RHODO), *Isochrysis galbana* (TISO), and *Prorocentrum minimum* (EXUV) in light and dark incubations. Ciliates were transferred from cultures grown on PLY429 into six-well plates containing each food at subsaturating levels (c. 100 cells/ml) and acclimated in the light at 19°C for 3 days. At the end of that time, the ciliates showed signs of color change (e.g., red for RHODO and brown for EXUV and TISO), but still contained some green chloroplasts. Ciliates were transferred to new wells containing the same algae they were acclimated to, at 10^4^ cells ml^−1^. For each food, there were two wells containing ciliates and one control well with algae only to verify that feeding took place. For each food treatment, we made two plates. One was incubated in the dark (wrapped in two layers of aluminum foil), and the other in the light (50 μmol m^−2^s^−1^). After 3 days, all wells were preserved with Lugol's iodine and ciliates and algae were counted.

### Long-term light/dark growth without acclimation

A second experiment to evaluate heterotrophic growth on EXUV differed in that it did not include an initial acclimation period so that the transition from one food type to another could also be evaluated for light- and dark-incubated cells. This experiment also extended for a longer period, to evaluate sustained heterotrophic (dark) growth. Ciliates were transferred from PLY-grown cultures into triplicate wells containing EXUV at 10^3^ cells ml^−1^ and incubated under light or dark conditions at 19°C. Food was added after 2 days to maintain saturating concentrations; after 5 days, five ciliates from each well were transferred to new wells at the same food and light conditions. The remaining ciliates were preserved and counted. This procedure was repeated at four successive intervals, whose lengths were 4, 4, 6, and 7 days, respectively. After 26 days, two of the dark wells suffered 100% mortality, so fresh triplicate light and dark wells were made from the survivors in the third dark well and followed for another 4 days.

### Food switching experiment

To evaluate survival and growth during the transition from light to dark, we performed a third experiment using the two algae that have consistently supported long-term culture of *S. rassoulzadegani*. Ciliates grown in the light for >3 weeks on RHODO or PLY429 were transferred to wells containing the other alga or an equal mixture of the two and incubated for 2 days under either light (50 μmol m^−2^s^−1^, 12:12) or dark conditions. Growth was measured by changes in abundance. Differences in responses to changes in food under light or dark conditions were evaluated by non-parametric Two-Way ANOVA performed on ranks (Sokal and Rohlf, 1995).

### Carbon and chlorophyll content

Cell volumes of ciliates and algae were calculated from measurements of linear dimensions of 10 cells each. Ciliate and algal carbon contents were estimated using volume to carbon relationships from Menden-Deuer and Lessard (2000). To measure chlorophyll content, *S. rassoulzadegani* cells were grown to high concentrations on a diet of PLY429 at concentrations that were saturating for growth (>10^4^ cells ml^−1^). We then separated the ciliates from their algal food using the ciliate's phototaxis. Ciliates were collected on glass fiber filters (Whatman GF/F) and extracted overnight in 90% acetone. Chlorophyll content was calculated from fluorescence.

## Results

### Isolation and cultivation

To date, we have isolated *S. rassoulzadegani* from tidepools in Scotland, CT, USA, and ME, USA. We also isolated it from shallow water at a beach near Sao Sebastiao, Brazil. The initial isolate, from Connecticut, was grown on swarmer cells (zoospores and/or gametes) from the green macroalga *Ulva sp*., but we have subsequently cultivated it mostly on the small green alga *Tetraselmis chui* (strain PLY429). It also grows well on the cryptophyte *Rhodomonas lens* (strain RHODO). Identity of all isolates was verified by amplification and sequencing of a portion of the ITS region of the ribosomal gene (Katz et al., 2005; McManus et al., 2010).

We compared growth on different foods and among different isolates using numerical response data (intrinsic growth rate vs. food concentration) fit to a Michaelis–Menten function with an added parameter to account for threshold feeding at low concentrations. A summary of 16 experiments on five different isolates is given in Table 1. In all cases, the food was the same (PLY429), and experiments were conducted at 19°C and 100 μmol m^−2^s^−1^ light on a 12:12 light: dark cycle. Maximum growth rates averaged 1.32 ± 0.42 (sd) d^−1^. The half-saturation (K) and threshold (t) parameters averaged 2300 and 440 cells ml^−1^, respectively, but were more variable, with coefficients of variation >100%. This is likely due in part to the difficulty of estimating parameters where the curve is changing steeply (K) or where food concentrations are very low (t).

**Table 1:** **Means and standard deviations for three parameters of the modified Michaelis-Menten equation fit to the data on growth vs. food concentration**.

**Parameter**	**Units**	**Mean**	**SD**	***n***
μ max	d^−1^	1.32	0.42	16
*K*	cells ml^−1^	2333	2747	16
*t*	cells ml^−1^	434	633	16

The data are from 16 experiments conducted on four isolates, from New England USA (three isolates), Brazil, and Scotland. In all cases, the food organism was Tetraselmis chui (PLY429).

**Figure 1:**
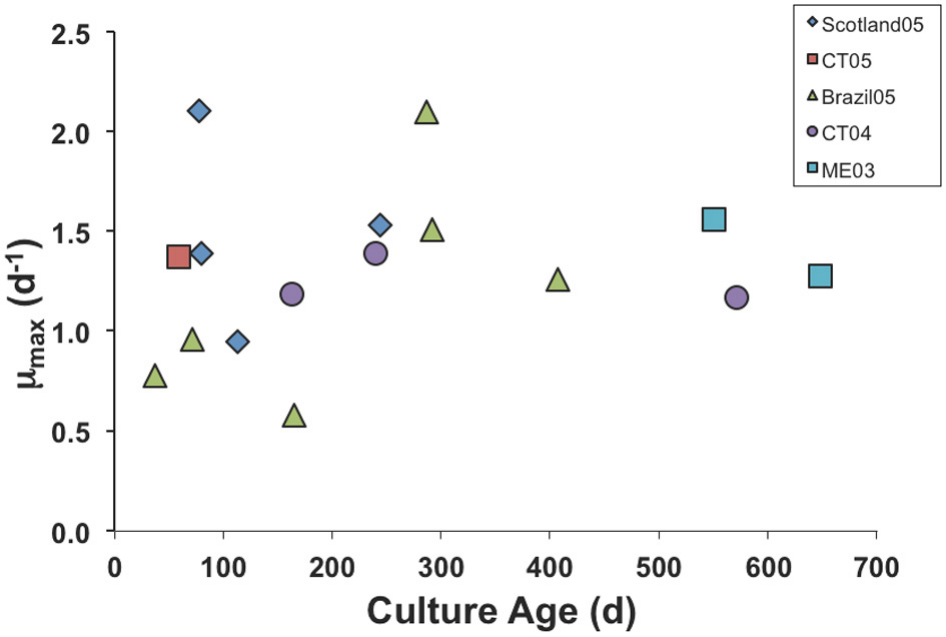
**Maximum growth rate parameter in numerical response curves for various isolates of *S. rassoulzaegani* fed *Tetraselmis chui* (PLY429), as a function of time since isolated.** Strain designations indicate isolation locale (CT, Connecticut USA; ME, Maine USA) and year of isolation.

We have had some isolates in culture for >3 years, but eventually all of the cultures die out, probably due to accumulated genetic defects and genetic drift in small laboratory populations (Bell, 1988; Montagnes et al., 1996). However, we found no clear trend of decline in μ_max_ with culture age. Even after 2 years in culture, our Maine (ME03) isolate grew at the same maximal rate as a freshly isolated one from Connecticut (CT05; Figure 1).

*Strombidium rassoulzadegani* grows very well on the green alga PLY429 and the cryptophyte RHODO (Table 2). It can be maintained for days to weeks on some other algae but we have never successfully cultured it for longer periods on them. When fed the dinoflagellate EXUV, or the prymnesiophyte TISO, the ciliate can be seen to ingest these cells and grow, gradually losing its green chloroplasts. After a few weeks at most, however, the cultures inevitably die out. A mixture of EXUV and TISO did not improve survival, and we never achieved growth on diatoms. Because the ciliates have been observed in field collections with green chloroplasts, we tried several other algae with green or yellow-green chloroplasts, including the tidepool-dwelling *Dunaliella tertiolecta* (DE) and the Eustigmatophyte *Nannochloropsis sp*. (UTEX2341), but could not achieve reliable growth (Table 2).

**Table 2:** **Ability of different algae to support growth in the oligotrich ciliate *Strombidium rassoulzadegani***.

**Algal food tested**	**Strain**	**Class**	**Result**
*Tetraselmis chui*	PLY429	Chlorodendrophyceae (Chlorophyta)	excellent growth; L >> D
*Rhodomonas lens*	RHODO	Cryptophyceae	excellent growth; L >> D
*Prorocentrum minimum*	EXUV	Dinophyceae	inconsistent growth, but L ≤ D
*Isochrysis galbana*	TISO	Prymnesiophyceae	inconsistent growth L, no growth D
*Thalassiosira pseudonana*	3H	Bacillariophyceae	no growth L or D
*Thalassiosira weissflogii*	Tweis	Bacillariophyceae	no growth L or D
Nannochloropsis sp.^*^	UTEX2341	Eustigmatophyceae	no growth, only L tested
*Stichococcus bacillaris*	StichoGSB	Trebouxiophyceae (Chlorophyta)	no growth, only L tested
*Chlorella autotrophica*	580	Trebouxiophyceae (Chlorophyta)	no growth, only L tested
*Dunaliella tertiolecta*	DE	Chlorophyceae (Chlorophyta)	no growth, only L tested
mixed diet	TISO + EXUV	Dinophyceae + Prymnesiophyceae	no growth, survival c. 1 week, only L tested

Class designations follow www.algaebase.org. Strain designations are from the Milford Microalgal Culture Collection. L, light (12:12 cycle, ~100 μmol m^−2^s^−1^); D, dark.

* Also called Chlorella minutissima, but see (Gladu et al., 1995).

**Figure 2:**
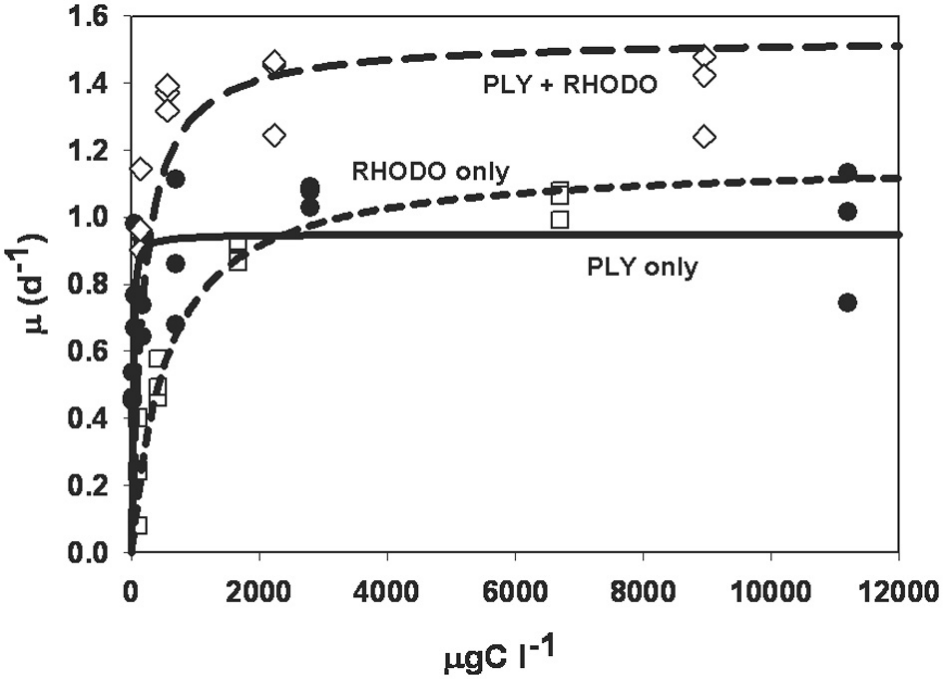
**Numerical response curves for *Strombidium rassoulzadegani* cultured on the chlorophyte PLY429 and transferred to a diet of PLY429, the cryptophyte *Rhodomonas lens* (RHODO) or a mixture of the two.** Curves are fit to the Michaelis-Menten function μ = μ_max_ [C−t]/(K + [C−t]), where μ is per capita growth rate, C is food concentration, K is the half-saturation constant, and t is a threshold below which growth is <0. For all three diets in this case, thresholds were not distinguishable from zero. The RHODO-only diet had a higher K than PLY-only or the mixed diet. In this case, the mixed diet appeared to give a higher maximum growth rate than either sole diet.

The numerical responses of ciliates grown on PLY, RHODO, or mixed RHODO/PLY were similar (Figure 2). In this experiment, values for the threshold parameter were not distinguishable from zero and K, the concentration where growth is half its maximum, ranged from 11 to 556 μgCl^−1^. μmax was highest on the mixed food at 1.54 d^−1^, but these parameters can be variable, and the difference was not statistically significant (Table 1). One feature of this experiment that is consistent in comparisons of these two foods is that the ciliate saturates more quickly on PLY429, suggesting that it only needs a small supply of fresh chloroplasts to maintain maximum growth rate on this food.

**Figure 3:**
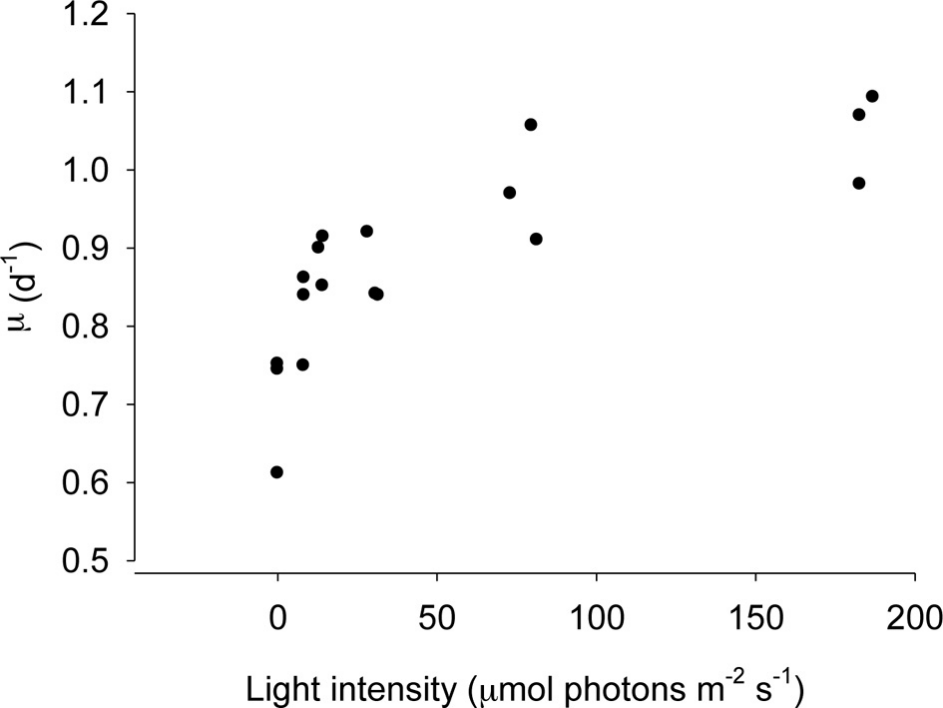
**Growth of *Strombidium rassoulzadegani* as a function of light intensity.** Ciliates were grown at 100 μ mol m^−2^s^−1^ and transferred to different light intensities without acclimation, ensuring that all chloroplasts were the same age, at a saturating food level of 2.5 × 10^4^ PLY429/ml.

The numerical response of *S. rassoulzadegani* to variations in light intensity is shown in Figure 3. Because acclimation at low intensities would have resulted in differential aging of the chloroplasts (ciliates acclimated at subsaturating light would have contained older chloroplasts due to slower growth), we transferred the ciliates from exponentially-growing cultures at high food concentrations directly into the experimental wells. Thus, there was likely a carryover based on previous food conditions, and positive growth at zero light probably indicates this. The data show that food-replete ciliate growth becomes light saturated at approximately 50 μmol m^−2^s^−1^.

### Carbon and chlorophyll content

*S. rassoulzadegani* has about 60 times the carbon content of its algal food (PLY429), but more than 100 times the chlorophyll content (Table 3). When cells were picked individually, rinsed in FSW, and exposed to ^14^C bicarbonate, they took up inorganic carbon in the light at rates per unit chlorophyll that were about half that of the algae. Because of the higher chlorophyll content, however, the turnover of C in ciliate and alga were about equal (0.13 and 0.14 d^−1^, respectively; Table 3).

**Table 3:** **Volume, carbon and pigment contents, and photosynthetic incorporation of ^14^C into the ciliate *Strombidium rassoulzadegani* and its food, *Tetraselmis chui* PLY429 (±standard deviation, where indicated)**.

	**Volume μm^3^ cell^−1^**	**ngC cell^−1^**	**pg chla cell^−1^**	**pgC cell^−1^h^−1^**	**pgC pg chl^−1^h^−1^**	**d^−1^**
*S. rassoulzadegani*	33,510 ± 2800	6.50 ± 0.53	137 ± 19	35.9 ± 6.6	0.26 ± 0.05	0.13 ± 0.02
*T. chui* PLY429	781 ± 199	0.112 ± 0.03	1.33 ± 0.72	0.67 ± 0.17	0.50 ± 0.13	0.14 ± 0.04

### Short-term light/dark growth after acclimation

**Figure 4:**
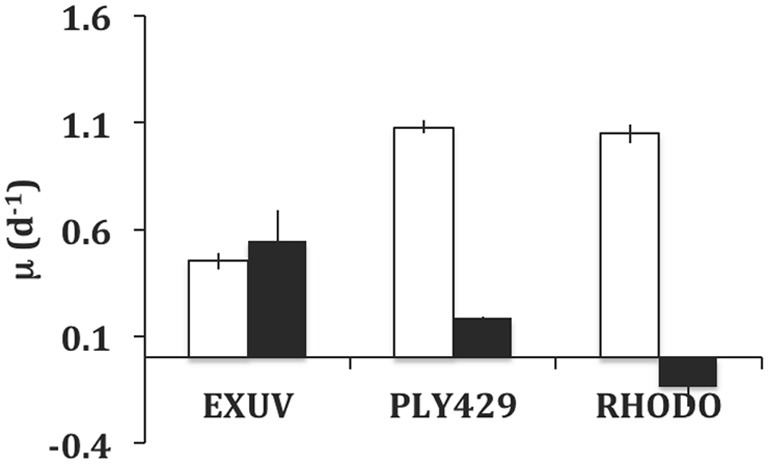
**Comparison of growth in light (12:12 cycle) vs. dark (continuous) for *Strombidium rassoulzadegani* fed one of three microalgae: *Prorocentrum minimum* (EXUV), *Tetraselmis chui* (PLY429), or *Rhodomonas lens* (RHODO).** Error bars are ranges of duplicates.

In the experiment in which ciliates were acclimated in the light to TISO, EXUV, PLY429, or RHODO for 3 days, then transferred to light and dark treatments for 3 days of growth, only the cells on TISO showed 100% mortality. The other three algae supported growth (Figure 4) and there was net grazing of all algae (higher algal net growth in ciliate-free controls), including TISO, under both light and dark conditions. Ciliates on PLY429 showed much higher growth in light, compared to the dark treatment, and on RHODO showed net mortality in the dark, compared to the light, where growth was the same as on PLY429. The ciliate grew less well on the dinoflagellate EXUV than on the other two algae in the light, but better in the dark, with no difference between light and dark treatments.

### Long-term light/dark growth without acclimation

**Figure 5:**
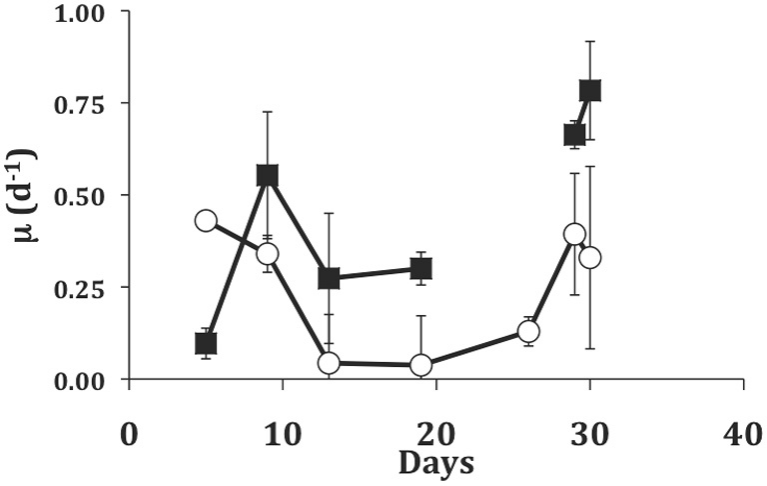
**Growth of *S. rassoulzadegani* on a diet of the dinoflagellate *Prorocentrum minimum* (EXUV) when incubated in dark (closed squares) or light (open circles).** Symbols represent means of growth rate in the preceding interval, with standard deviations of triplicates. The gap in the dark treatment occurred when two of three replicates suffered 100% mortality, so the last two points in the dark treatment are ciliates transferred from the surviving well.

In the longer term experiment with the dinoflagellate EXUV as food, we were able to grow *S. rassoulzadegani* for 30 days (Figure 5). During the initial transition from PLY-grown culture (5-days), the ciliate showed higher growth in light than in the dark, probably due to the fact that it was still using PLY429 chloroplasts. From day 5 to day 10, however, growth in the dark increased and growth in the light declined. Growth remained higher in the dark for the remainder of the experiment except that two of the three dark replicates suffered 100% mortality between days 19 and 26. After 26 days, the ciliates in the light treatment increased their growth rates, but growth was still slower than in the dark.

### Food switching experiment

**Figure 6:**
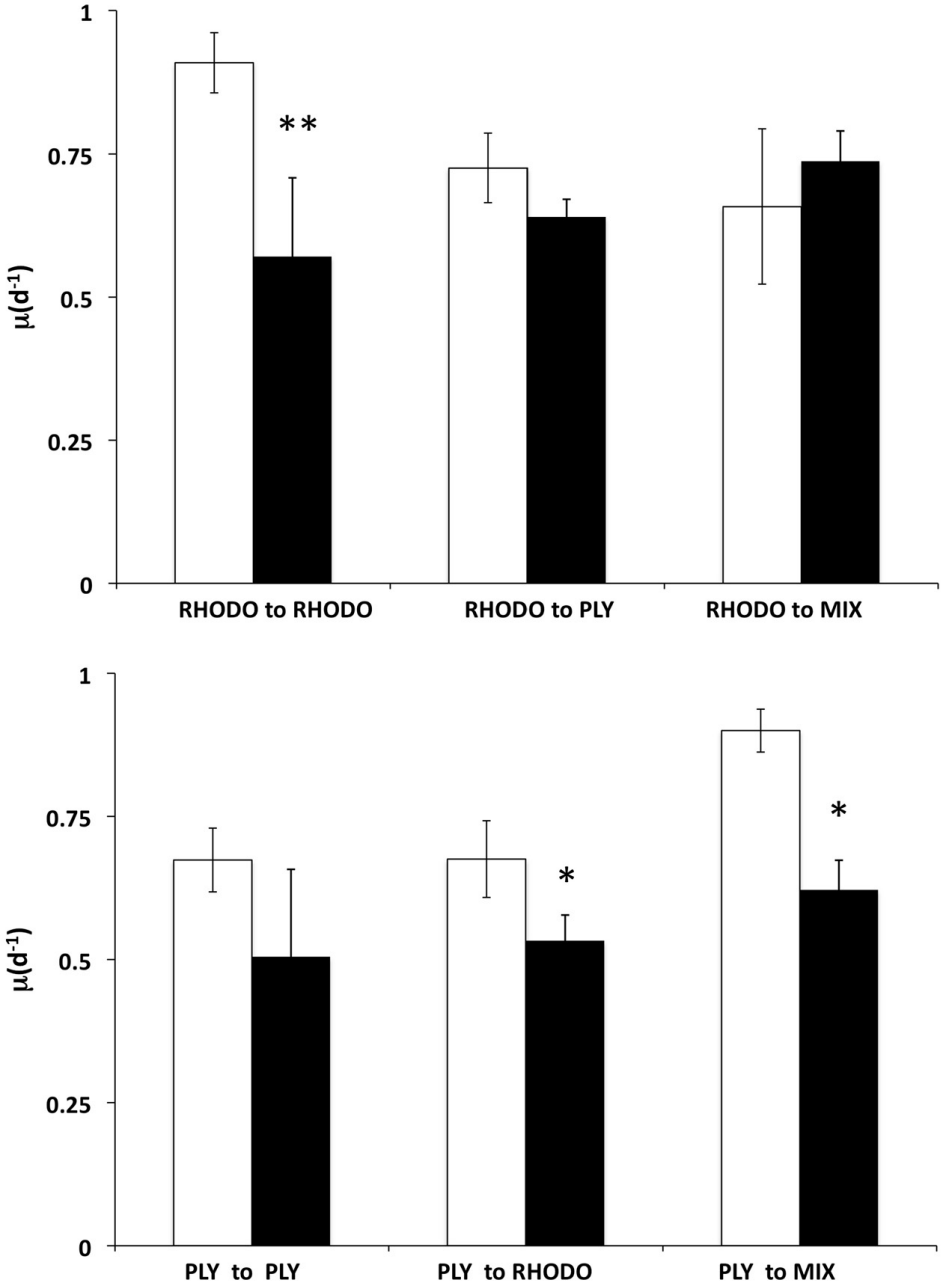
**Effect of switching food sources on light vs. dark growth.** Ciliates cultured on *Rhodomonas lens* (RHODO, top panel) were transferred to RHODO, PLY, or an equal mixture of the two. Ciliates cultured on *Tetraselmis chui* (PLY, bottom panel) were likewise transferred to the PLY, RHODO, or a mixture. Means with standard deviations of triplicates. Significant differences between light and dark treatments indicated by ^*^ and ^**^ for *P* < 0.05 and 0.01 (*t*-test), respectively.

We examined the growth responses of the ciliates to the transition between its two preferred foods, PLY429 and RHODO, and between light and dark conditions, during a 2 day experiment (Figure 6). Ciliates grown on RHODO grew better in light than in dark when kept on RHODO, although there was apparently a carryover effect that allowed about one division in the dark. This is supported by the fact that cells in the dark treatments were significantly smaller than those in the light (19% and 43% less cell volume in the PLY to PLY and RHODO to RHODO treatments, respectively), even though they had similar division rates. When switched from RHODO to PLY, there was no difference between light and dark treatments. Non-parametric Two-Way ANOVA indicated no significant added variance for the light or food treatments, but there was a significant interaction effect of light × food (*P* < 0.05). For the ciliates grown on PLY429, growth was higher in light than in dark for all three foods (*P* < 0.01 for the light effect), but there was no effect of food treatment on growth, and no significant interaction. As in the previous experiment, growth rate increased in the transition from PLY429 to the mixed diet. The RHODO-grown cells did not increase growth rate on the mixed diet (Figure 6).

### Phototaxis

Ciliates that were cultured on PLY429 and transferred for 5d to RHODO, EXUV, or TISO all showed strong positive phototaxis. Regardless of food alga, all five replicates had at least 7 out of 10 ciliates migrate into the light within 2 min. Assuming that non-phototactic cells would be randomly distributed in the capillaries and hence equally likely to be in the dark or the light, the probability of this result occurring by chance is 2^−5^, or 0.031. Even after several weeks of cultivation on RHODO, during which the ciliate lost its eyespot pigments, strong positive phototaxis was still retained (data not shown).

## Discussion

We originally discovered *S. rassoulzadegani* as a sequence of the ITS region of the ribosomal gene (ITS1-5.8S-ITS2) from a clone library of samples collected in tide pools on the Irish Sea and Galway Bay, Ireland (clade vii in Katz et al., 2005). In these field-collected samples of green tidepool ciliates, we found surprising hidden diversity, with sequences differing by up to 16% at this locus (Katz et al., 2005). Subsequently, we isolated and cultivated clade vii from samples collected in Scotland, Brazil, and several sites in New England, USA. After cytological staining and comparision with previously-reported ribosomal gene sequences in GenBank, we named clade vii as the new species *S. rassoulzadegani* (McManus et al., 2010).

*S. rassoulzadegani* is typical of mixotrophic oligotrichs in requiring a constant supply of fresh chloroplasts from ingested food in order to maintain photosynthetic capability. Complete turnover of chloroplasts takes about 48 h (Schoener and McManus, in review). *In situ*, it has green chloroplasts, like its congener *S. oculatum* and several other estuarine strombidiids (Jonsson, 1987; Stoecker et al., 1988a), and it likely feeds on the gametes or zoospores of green macroalgae (McManus et al., 2004).

In common with several other “green” strombidiids, *S. rassoulzadegani* has an apical eyespot that is likely used in phototaxis. Because both the putative natural prey (swarmer cells of green macroalgae) and green microalga on which we cultivated it contain eyespot pigments and a photosensing system, we initially speculated that the source of the ciliate eyespot was also its food. While it appears that ciliates cultivated on the cryptophyte RHODO do lose their eyespots, their ability to orient toward light in a positive phototaxis was not diminished in our observations. There may be some shading of the ciliate photoreceptor (unknown at present) by the sequestered chloroplasts to compensate for eyespot loss, but this is speculative. In the context of mixotrophy's costs and benefits, the potential role of algal eyespot pigments in the ciliate's phototaxis deserves further study.

This ciliate grows equally well on the green PLY429 or the red RHODO (Figure 2), retaining both kinds of chloroplasts. It has been maintained for >3 years in the lab on PLY429. We did not observe any decline in physiology (as indicated by maximum growth rate) in long term isolates, though most of them die out within 2 years for reasons that are not known. Although use of cryptophyte chloroplasts has been shown in several other mixotrophic oligotrichs, it seems unusual that they would be interchangeable with those of green algae insofar as ciliate growth rate is concerned. The transition from red to green chloroplasts over 2d was nearly indistinguishable from that of green to red (Figure 6), except that RHODO-grown cells showed a significant interaction between food and light treatments when exposed to a new food (ANOVA results; Figure 6), whereas PLY-grown cells maintained about the same growth advantage in light vs. dark regardless of food (Figure 6). Given the very strong differences between light and dark growth on RHODO (Figures 4, 6), we interpret this to indicate that ciliates switching from RHODO to another kind of chloroplast, in this case PLY429, suffer some disadvantage in comparison to PLY-grown cells, which retain their PLY429 chloroplasts longer when transferred to RHODO (Schoener and McManus, in review). This suggests a stronger integration of green chloroplasts when the ciliates are feeding on green algae. More observations of growth rate and growth efficiency in light and dark at low food concentrations may help to resolve this issue.

On either of these food organisms, *S. rassoulzadegani* appears to be a “persistent/obligate” mixotroph, *sensu* Stoecker et al. (2009), requiring both food and light for growth (Figure 4). It can, however, grow for days or weeks on some other algae, including the dinoflagellate EXUV (Figures 4, 5). This is much longer than the period in which it can retain its green chloroplasts. Furthermore, it can grow as well or better in the dark compared to in the light on EXUV, suggesting that growth is fully heterotrophic. The transition from mixo- to heterotrophic growth sometimes fails, however, and there may be some conditions of food or ciliate growth cycle that prevent it. After several weeks of feeding on EXUV in the dark, growth rates were about 0.75 d^−1^, similar to those of non-mixotrophic oligotrichs (0.5–1.4 d^−1^; Gismervik, 2005). These results thus suggest caution in assigning “obligate” status to this kind of symbiotic arrangement unless exhaustive trials on other foods have been performed.

One interesting observation from the experiment in which *S. rassoulzadegani* was maintained for 30d on EXUV was that ciliates initially grew better in the light, undoubtedly due to the leftover chloroplasts from PLY429, but then grew much worse than the dark treatments, just surviving in the light (Figure 5). After about 2 weeks, growth rates in the light increased, but remained lower than those in the dark despite the fact that in the light treatment food algae were more abundant than in the dark treatments due to increased algal growth in the light. It may be that exposure to light suppresses digestion of the chloroplasts. Laval-Peuto and Febvre (1986) and Stoecker and Silver (1990) observed vacuoles containing deteriorating chloroplasts. These appeared to be autophagous and distinct from food vacuoles. They also were rare, suggesting that digestion of chloroplasts usually does not occur and old, non-functional chloroplasts may be egested rather than digested. We have not observed this directly, and starved ciliates appear to retain chloroplasts right up until death (Schoener and McManus in review). If the EXUV chloroplasts in this case are unsuitable for phototrophic use by the ciliate and somehow marked as immune from digestion in the light, they would become a liability and slow ciliate growth relative to that in the dark until they could be egested. We have not attempted to measure ^14^C incorporation by ciliates grown on EXUV, but we speculate that those chloroplasts are not usable by the ciliate and hence slow its growth in the light. Much more work remains to be done on the intracellular signals that prevent digestion of chloroplasts in mixotrophic ciliates before this issue can be resolved.

The observation of slower growth in the light on EXUV raises the question of the benefits and potential costs of mixotrophy. It seems obvious that the ability to retain and use ingested chloroplasts is a strong benefit to the ciliate because photosynthetically-incorporated carbon would be an autotrophic subsidy for growth. In culture, mixotrophs like *S. rassoulzadegani* need relatively little food to achieve maximum growth (Figure 2) and have high growth efficiencies (Johnson and Stoecker, 2005; Schoener and McManus, in review). Dolan and Perez (2000) found evidence of reduced predation on mixotrophs, compared to heterotrophic ciliates, suggesting a second benefit of mixotrophy. Berger (1980) observed a similar phenomenon in *Didinium/Paramecium* predation, with algal symbiont-bearing *Paramecium* being half as susceptible to predation as those without symbionts. Other potential benefits, including removal of nitrogenous wastes or provision of oxygen, have been demonstrated in other symbioses (e.g., Finlay et al., 1996; Davy et al., 2002), but have not been documented yet in plastid-sequestering ciliate mixotrophs, to our knowledge.

The potential costs of mixotrophy are not well-known. In ciliates where mixotrophy is obligate, the light requirement obviously limits them to the photic zone. However, the observation of “obligate” mixotrophy in chloroplast-retaining ciliates has been based mostly on laboratory cultures, where diversity of food is limited. Studies on field-caught mixotrophs have revealed multiple chloroplast types in some species (Laval-Peuto and Febvre, 1986; Stoecker and Silver, 1987), and it seems likely that the diets of mixotrophs in nature are varied. Our finding that a ciliate that is apparently obligate when fed one alga could be maintained in the dark on another suggests that some switching between mixotrophy and heterotrophy may be possible, at least in this species and under some circumstances. The fact that heterotrophic growth was worse in the light suggests that ciliates with the capability for chloroplast sequestration may pay a price in growth when suitable chloroplasts are not available in the diet. Other possible costs of retaining chloroplasts, such as the increased oxidative stress due to symbionts that has been observed in zooxanthellae-bearing coral larvae (Yakovleva et al., 2009), have not been studied in mixotrophic ciliates and deserve investigation.

### Conflict of interest statement

The authors declare that the research was conducted in the absence of any commercial or financial relationships that could be construed as a potential conflict of interest.
